# Participatory systems mapping: a review of population health research practice

**DOI:** 10.1186/s12961-026-01457-6

**Published:** 2026-03-10

**Authors:** Carolyn Blake, Benjamin P. Rigby, Martin White, Kirstin R. Mitchell, Sharon A. Simpson, Nigel Gilbert, Roxanne Armstrong-Moore, Petra S. Meier, Alexandra Penn, Mohammad Hassannezhad, Valerie Wells, Laurence Moore

**Affiliations:** 1 School of Health and Wellbeing, University of Glasgow, Glasgow, United Kingdom; 2 Population Health Sciences Institute, Newcastle University, Newcastle, United Kingdom; 3 MRC Epidemiology Unit, University of Cambridge, Cambridge, United Kingdom; 4 Centre for the Evaluation of Complexity Across the Nexus, University of Surrey, Guildford, United Kingdom; 5 Department of Informatics, University of Sussex, Brighton, United Kingdom; 6 Centre for Systems Engineering, University College London, London, United Kingdom

**Keywords:** Participatory systems mapping, Systems thinking, Population health, Involvement, Causal loop diagram, System dynamics, Fuzzy cognitive mapping, Bayesian belief networks, CECAN PSM, Systems-based theory of change

## Abstract

**Background:**

Participatory systems mapping (PSM) methods are increasingly applied in population health research to understand and address complex challenges. Despite their growing use, there remains limited understanding of how these approaches are implemented in practice. This systematic scoping review aimed to explore the application of PSM in population health research, identify methodological gaps and highlight opportunities for advancing methods development and reporting standards, with particular attention to participatory approaches.

**Methods:**

A systematic search of OVID MEDLINE and Scopus identified peer-reviewed papers published in English between January 2000 and September 2023 that: (1) applied and presented the results of PSM related to population health or health improvement questions and (2) incorporated a participatory design. Two reviewers screened and assessed papers, extracting data on study characteristics, participatory approaches, map features and integration of conceptual frameworks and methods not directly related to PSM.

**Results:**

In the 123 included studies, involving stakeholders in building causal loop diagrams was the most commonly used approach. Variability was evident in geographical focus, study design, application and reporting. Participant involvement was mostly limited to map building, with less engagement in map validation. Significant gaps in reporting study samples and procedures were identified. A small number of studies involved end users or people with lived experiences in mapping processes. Only a few studies evaluated stakeholders’ experience with participatory processes. Lessons learnt on participatory processes include: PSM in population health benefits from cross-disciplinary, inclusive collaboration and capacity-building efforts that support meaningful involvement, shared ownership and trust among diverse stakeholders. Adaptability in the design of PSM approaches, continuous reflection and long-term partnerships are essential to maintaining relevance, enhancing impact and fostering systemic change over time.

**Conclusions:**

To advance participatory systems mapping in population health, there is a need for further methodological innovation, stronger stakeholder engagement and more transparent, reflexive reporting practices. Building capacity through training, practical guidance and cross-disciplinary communities of practice will also be essential to support rigorous and inclusive application of these methods.

**Supplementary Information:**

The online version contains supplementary material available at 10.1186/s12961-026-01457-6.

## Background

Population health challenges are complex, shaped by determinants spanning diverse domains. Recent years have seen a rise in the use of systems thinking in research, policy and practice to help make sense of complexity [1]. This includes the use of participatory systems mapping (PSM) [2] – a term encompassing methods that “engage stakeholders with varied knowledge and perspectives in creating a visual representation of a complex system to explore, and document perceived causal relations between elements in the system” [3]. A system can be defined as a set of interconnected elements within a physical or conceptual boundary (e.g. a youth centre, a food system, a country) [4, 5].

Participatory systems mapping offers a valuable tool to explore population health challenges, potentially revealing their complexities and dynamics. It also facilitates the integration of systems thinking into policy-making [6].

To date, two scoping reviews have explored the use of PSM in population health. These were focused on a specific methodological approach in public health research (Baugh Littlejohns et al., 2021, on the use of causal loop diagrams) or topic (van den Akker et al., 2023, on the application of PSM to noncommunicable disease risk factors). However, multiple different systems mapping methods exist, each with different characteristics and suitable scenarios for use. A broader understanding of their application, strengths and weaknesses will guide future development, use and reporting.

We undertook a systematic scoping review of peer-reviewed literature to inform the development of guidance on the selection and design of participatory systems mapping methods in population health contexts [3]. The review aimed to (1) understand the breadth of applications of participatory approaches to causal system mapping methods in research, (2) critically examine their implementation and reporting and (3) identify emerging trends, gaps and opportunities within the field.

## Methods

### Study design

This systematic scoping review was guided by the Preferred Reporting Items for Systematic Reviews and Meta-Analyses extension for scoping reviews (PRISMA-ScR) [9]. A scoping review is appropriate to the aim of critically exploring the characteristics of an emerging field in population health [10].

### Eligibility criteria

Included articles: (i) were peer-reviewed; (ii) published in English between 1 January 2000 and 30 September 2023 (2000 selected owing to limited earlier work in this area prior to this date); (iii) reported on PSM in the population health/health improvement context; (iv) incorporated a participatory design; and (v) included system maps depicting *open* social systems. Exclusion criteria were as follows: studies reporting on *closed* systems – that is, systems with limited or no interaction with external contextual influences (e.g. studies conducted within a single hospital ward, medical education setting, workforce planning, or health administration/management). These studies were excluded because the focus of this review was on open, population-level systems, showing interactions with broader social, economic and environmental contexts. We also excluded grey literature, protocols and reviews. Population health was defined as the health outcomes of a group of individuals, including the distribution of such outcomes within the group [11], determinants of health and interventions aimed at addressing these determinants and outcomes. Participation was defined as the involvement of at least one stakeholder external to the research team in the map building/validation process.

### Search strategy

A search strategy for two electronic databases (OVID MEDLINE and Scopus) was developed in consultation with an information scientist (V.W.). Searches contained terms related to “population health”, “system map” and “participatory” methods (see Additional File 1 for full search strings). Two previously published population health-related search strings [12, 13] were used to inform search terms. Additional terms were added to reflect the global focus of the current review, and population health research terminology in use outside the United States. We also excluded search terms related to medical education, workforce planning and health administration/management (as these related to closed systems; see eligibility). Additional articles were identified by searching reference lists of excluded review articles and forward citation chaining of excluded protocols (note, citation chaining was not performed on included publications). Further articles known to the authors were also included. All articles were imported into Covidence [14] to support the review process. Initial searches were conducted in June 2021 and updated in October 2023.

### Selection process

Duplicate articles were identified and removed using Endnote and Covidence software. Article selection was then piloted by two reviewers (C.B. and B.R.) using an established process [15]. Thirty papers, selected at random using Endnote and Randomizer.org [16], were independently screened in each pilot round. Inter-rater agreement for study inclusion was calculated using percent agreement [15], with screening proceeding once agreement exceeded 75%. This threshold was reached after two rounds of pilot testing (60 papers in total).

Title and abstract screening were conducted independently by two reviewers (C.B. and B.R.), and articles included by at least one reviewer were taken forward. Full-text screening was then performed in duplicate by two of three authors (C.B., B.R. and R.A.-M.), and by C.B. and B.R. for the updated search. Discrepancies were discussed and resolved by consensus at each stage (including clarification sought from authors of two studies).

### Data extraction

Information from each included paper was extracted using Covidence into a data extraction sheet, including study characteristics, participatory approach to mapping, map features and properties, and integration of other conceptual frameworks and methods not directly related to PSM approaches. Extraction categories were developed iteratively during the piloting of full-text screening, and in consultation with systems mapping experts (Additional File 2). Extraction was piloted with two papers, and subsequently conducted independently by C.B., B.R. and R.A.-M. for the initial search, and by C.B. and B.R. for the updated search. A sense-checking meeting was held to discuss uncertainties and finalise data extraction.

### Data analysis

Data analyses were conducted by C.B. and B.R. A framework was developed by all authors to present the literature according to PSM methods and extraction headings cited above. Count data were used in relation to each of the key framework dimensions to identify trends in the use of PSM methods in population health, while textual data were used to contextualize the methodological and research landscapes. Informed by the Framework Method [17], we also carried out a qualitative synthesis of text presenting the authors’ reflections on the participatory approach used in systems mapping. One researcher (C.B.) analysed the extracted data using an inductive coding process to identify common concepts and patterns in the data. The resulting codes were subsequently consolidated into overarching themes that represented the main lessons learnt highlighted in the included studies.

## Results

### Search results

**Fig. 1:**
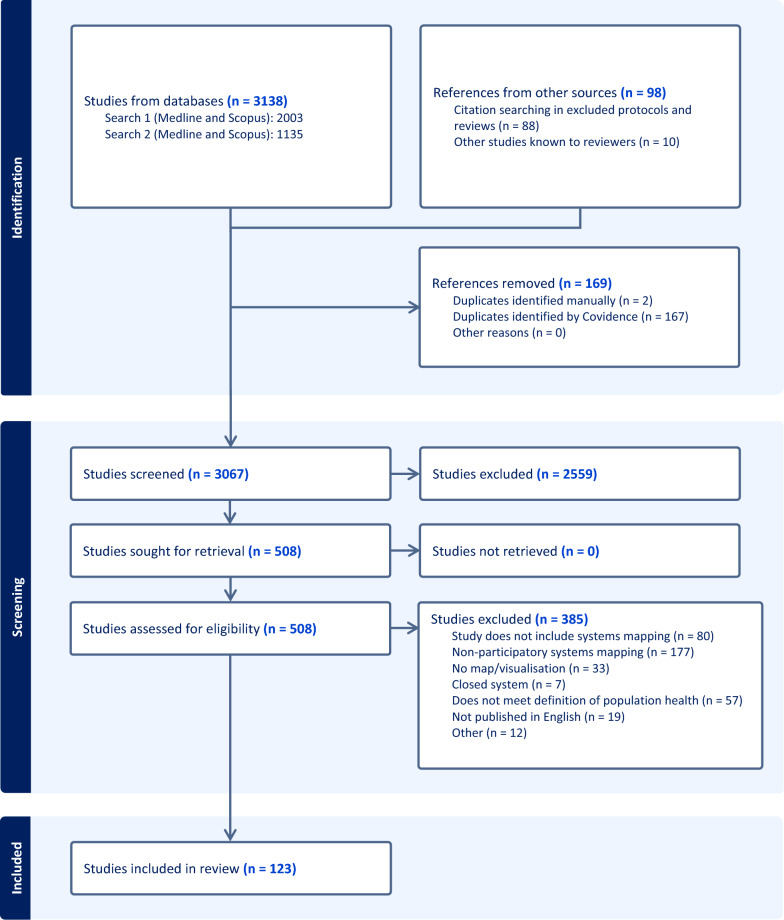
PRISMA-ScR flowchart

Electronic database searches identified 3138 records, while 98 records were identified via other methods. In total, 169 duplicate records were removed from Covidence. Following title and abstract screening of 3067 records, 508 articles were screened in full, and 123 met the criteria for inclusion in this review. Figure 1 shows the flow of records through the selection process.

### Study characteristics

A descriptive summary of included studies is presented in Additional File 3. Although our review included studies published from 2000 onwards, nearly half of the included studies (58 of 123) were published since 2020.

#### Context

Most studies were carried out in North America (*n* = 43) and Australia/Oceania (*n* = 33). The remaining studies were in Asia (*n* = 21), Europe (*n* = 14), Africa (*n* = 8) and South America (*n* = 1). Overall, participatory systems mapping took place in 40 countries and territories. The geographical context was unclear in four studies.

A majority of studies mapped systems in stable contexts (*n* = 107) (as defined by the Organisation for Economic Co-operation and Development (OECD)) [18], with 13 studies mapping systems in fragile settings, and 18 in settings with disturbances amid stable contexts (e.g. poverty, violence, marginalised Indigenous groups, extreme heat). This information was unclear in five papers.

In terms of spatial context, mapping at the local level was most common (*n* = 45), followed by national (*n* = 31), and subnational level (e.g. regional) (*n* = 27). We found six multicountry studies [19–24]. These spatial boundaries were unclear in 14 papers. Most studies mapped within specific spatial contexts (e.g. understanding an issue within a specific location) (88/123), whereas 32 studies mapped an abstract/generalised context (e.g. focusing on an issue in general terms, without a specific spatially located tie). This information was unclear in three studies (Table 1).

**Table 1:** Study contexts

Geographical areas (continents)	*N*	Citations
Europe	14	[24, 26, 27, 48, 51, 62, 72–79]
Africa	8	[49, 80–86]
Asia	21	[45, 46, 56, 57, 63–65, 87–100]
Australia/Oceania	33	[23, 30, 34, 38, 42, 43, 50, 52, 55, 60, 61, 101–117, 147–151]
North America	43	[19–21, 23, 25, 28, 29, 31, 33, 35–37, 39–41, 47, 58, 59, 118–142]
South America	1	[21]
Unclear/not reported	4	[143–146]

Stable/fragile settings	*N*	Citations
Stable settings	105	[19, 23–31, 33–43, 45–48, 50–52, 55–65, 72–79, 81–83, 87–90, 93, 95, 98, 100–142, 147–151]
Stable settings with *disturbances*	18	[19, 28, 30, 31, 39, 52, 81, 82, 87, 93, 100, 125, 128, 131, 132, 137, 138, 151]
Fragile settings	11	[49, 80, 84–86, 91, 92, 94, 96, 97, 99]
Mixed settings (stable and fragile)	2	[20, 21]
Unclear/not reported	5	[22, 143–146]

Spatial scope of mapping	*N*	Citations
Local	45	[28, 29, 31, 33, 36, 38, 40–43, 47, 50, 59, 63, 64, 73, 76, 78, 80, 84, 86, 107, 109, 112, 115, 116, 119, 121, 123, 124, 126–128, 131–136, 138, 140, 142, 145, 147, 148]
Subnational (e.g. regional)	27	[25, 30, 34, 39, 45, 46, 51, 52, 55, 56, 77, 81, 82, 87, 91, 95, 96, 101, 104, 110, 111, 113, 114, 118, 125, 137, 139]
National	31	[26, 35, 48, 49, 57, 60–62, 65, 74, 75, 79, 83, 88–90, 92–94, 97–100, 103, 106, 108, 122, 141, 146, 149, 150]
Multicountry	6	[19–24]
Global	0	N/A
Unclear/not reported	14	[27, 37, 58, 72, 85, 102, 105, 117, 120, 129, 130, 143, 144, 151]

Specific spatial context/abstract context	*N*	Citations
Specific spatial context	88	[19, 21, 23, 25, 26, 28–31, 33, 34, 36, 38–43, 45–50, 52, 55, 56, 59, 62–65, 73, 75–89, 91, 93–95, 99–101, 104, 107–116, 119, 121, 123, 125–128, 131–140, 142, 147, 148, 150, 151]
Abstract context	32	[20, 22, 24, 35, 37, 51, 57, 58, 60, 61, 72, 74, 90, 92, 96–98, 102, 103, 105, 106, 118, 120, 122, 129, 130, 141, 143–146, 149]
Unclear/not reported	3	[27, 117, 124]

#### Population health research areas

Participatory systems mapping was applied to a range of population health research areas. Most common were studies on food, nutrition or obesity (mainly in childhood) (*n* = 32); social determinants of health (*n* = 20); drug use or alcohol consumption (*n* = 17); communicable diseases (*n* = 10); noncommunicable diseases (*n* = 9); and mental health and wellbeing (*n* = 9). Table 2 lists all other population health areas.

**Table 2:** Population health area, methods, purpose and type of project

Population health research areas	*N*	Citations
Food, nutrition and obesity	32	[19, 24, 27, 31, 34, 41, 42, 50, 52, 59, 81, 104, 106–108, 110, 112, 113, 115–117, 123, 124, 126, 127, 138, 141, 144, 147, 148, 150, 151]
Social determinants of health	20	[21, 23, 33, 38, 39, 43, 45, 51, 58, 63, 64, 73, 84, 101, 119, 120, 133, 136, 137, 146]
Drug use or alcohol consumption	17	[25, 26, 35, 37, 46, 56, 57, 60, 77, 118, 122, 130, 139, 140, 142, 143, 149]
Communicable diseases	10	[40, 49, 62, 83, 92, 103, 111, 129, 132, 134]
Noncommunicable diseases	9	[29, 47, 55, 72, 74, 75, 91, 100, 125]
Mental health and wellbeing	9	[30, 79, 87, 96, 102, 121, 128, 135, 145]
Neonatal, child and adolescent health	5	[36, 80, 85, 86, 97]
Coronavirus disease 2019 (COVID-19)	5	[88, 89, 94, 105, 109]
Physical activity	4	[20, 61, 78, 114]
Health systems	6	[22, 48, 76, 93, 95, 99]
Maternal health	2	[65, 82]
Oral health	2	[90, 98]
Violence	2	[28, 131]

Methods	*N*	Citations
Causal loop diagram (CLD) (as a stand-alone method)	72	[19–24, 26–28, 33, 38, 40, 41, 45–48, 50, 52, 55, 57, 60–62, 72, 73, 78, 80–83, 86, 88–92, 99–109, 113–116, 118–121, 123, 124, 126–128, 136–143, 147, 148, 151]
Systems dynamics modelling (with CLD and/or stock and flow diagrams)	30	[30, 31, 35, 37, 39, 42, 43, 58, 65, 74, 76, 77, 84, 85, 95–98, 110, 112, 117, 129–135, 145, 149]
Stock and flow diagrams (stand-alone or with other method)	4	[37, 87, 93, 122]
Fuzzy cognitive mapping (FCM)	5	[29, 51, 59, 94, 125]
Bayesian belief networks (BBN)	2	[75, 111]
Centre for the Evaluation of Complexity across the Nexus (CECAN) PSM	1	[49]
Systems-based theory of change diagrams	2	[34, 56]
Other causal system maps	11	[25, 36, 63, 64, 79, 95, 110, 131, 144, 146, 150]

Purpose	*N*	Citations
Understanding of an issue or a context	52	[19–21, 27–29, 34, 40, 45–47, 51, 52, 57, 59, 72, 73, 80–83, 87–93, 101–109, 118–128, 143, 144, 147, 148]
Simulation modelling	35	[30, 31, 35, 37, 39, 42, 43, 48, 49, 58, 65, 74–77, 84, 85, 94–98, 110–112, 129–135, 145, 146, 149]
Leverage point(s) identification	21	[22, 24, 26, 38, 41, 50, 55, 60, 61, 63, 64, 86, 99, 113, 114, 136–140, 150]
Intervention/policy development or refinement	9	[23, 25, 33, 62, 78, 79, 100, 141, 151]
Monitoring and/or evaluation of interventions, policies or strategies	5	[36, 56, 115, 116, 142]
Research question identification	1	[117]

Type of project	*N*	Citations
Stand-alone systems mapping project	67	[20, 22, 23, 25, 26, 28, 30, 31, 35, 37–39, 42, 43, 45, 49, 52, 59, 62, 72–76, 78, 83, 84, 87–89, 91–95, 97–99, 102, 105, 108–114, 119–123, 128–130, 132, 133, 136, 138, 142–146, 148–150]
Part of a wider project	47	[19, 21, 24, 29, 33, 34, 36, 40, 41, 47, 48, 50, 51, 55, 60, 61, 63–65, 77, 79, 81, 82, 85, 86, 96, 100, 101, 103, 104, 106, 107, 115–118, 124–127, 131, 134, 135, 137, 139, 140, 147]
Unclear or not reported	9	[27, 46, 56–58, 80, 90, 141, 151]

#### Methods, purpose and type of project

We then examined study methods and the main purposes reported for their use (Table 2). In total, 91/123 papers included causal loop diagrams (CLDs). A majority of these used CLDs as a stand-alone method (*n* = 72), and as a foundation for systems dynamics (SD) modelling (*n* = 18). When a causal loop diagram was used as a stand-alone method, its origins in the system dynamics field were not always explicitly acknowledged. The use of stock and flow diagrams was also common (*n* = 28), most of which were included as a foundational step in SD modelling (*n* = 24). Few examples of participatory approaches to other methods were identified: fuzzy cognitive mapping (FCM) (*n* = 5), Bayesian belief networks (BBN) (*n* = 2), systems-based theory of change diagrams (*n* = 2), CECAN PSM (*n* = 1) and other causal maps (*n* = 11). See Additional file 4 for a brief description of these methods.

The most common purpose for using a PSM method was to refine the understanding of an issue or a context (52/123) or to underpin simulation modelling (e.g. using a SD model) (*n* = 35). Other common purposes were to identify leverage point(s) (i.e. a location in the system where a shift can produce important change) (*n* = 21); intervention/policy development or refinement (*n* = 9); monitoring and/or evaluation of interventions, policies or strategies (*n* = 5); and research question identification (*n* = 1). Systems mapping was conducted either as a stand-alone project (*n* = 67) or as part of larger studies (*n* = 47); this was unclear or not reported in nine papers (Table 2).

### Participatory approach

We reviewed studies for their approach to participation, including involvement of stakeholders in map building and map validation (Table 3). By map building, we mean the identification of factors and causal relations in a system. By map validation, we mean the process of assessing how well a map reflects a system as perceived by participating stakeholders. Alternatively (for quantitative mapping methods), this may also refer to the process of determining whether a simulation model reproduces target outputs [3].

**Table 3:** Participatory approaches

Multisectoral versus single sector	*N*	Citations
Participants from multisectoral backgrounds	102	[19–23, 25–31, 33–43, 45–47, 49–52, 55–58, 60–65, 72–74, 77–80, 82–87, 89–91, 93, 94, 96–98, 100, 101, 103, 105–107, 109, 110, 112–117, 120–124, 126–129, 131, 133–138, 140–148, 150, 151]
Participants from single sector/discipline	14	[24, 48, 75, 81, 92, 95, 99, 102, 104, 108, 111, 118, 125, 132]
Unclear/not reported	7	[59, 76, 88, 119, 130, 139, 149]

Participation by stage	*N*	Citations
Both map building and validation	60	[23, 25–27, 30, 31, 36–38, 40, 43, 46, 48–52, 60, 63, 64, 72, 74, 79, 82, 84–87, 90, 94, 98–101, 103–109, 112, 115–117, 119, 120, 122, 126, 129, 131, 135–138, 144, 145, 147–149]
Only map building	32	[19, 20, 24, 29, 33, 41, 47, 58, 59, 62, 65, 73, 75, 78, 83, 88, 91, 93, 96, 110, 118, 123–125, 127, 130, 134, 140, 142, 146, 150, 151]
Only validation	27	[21, 22, 34, 35, 39, 45, 55–57, 61, 77, 80, 81, 89, 92, 95, 97, 102, 111, 113, 114, 121, 128, 132, 139, 141, 143]
Unclear/not reported	4 19	Lack of clarity in all stages: [28, 42, 76, 133] Lack of clarity in one of the stages: [20, 24, 29, 33, 34, 41, 55, 57, 65, 78, 91, 93, 96, 118, 123, 124, 130, 134, 146]

Data collection method	*N*	Citations
Workshops	97	[19–21, 23, 24, 26–31, 33–36, 38–43, 45, 47–50, 52, 55, 58, 60, 62–65, 72, 73, 78, 79, 81–85, 87–93, 96, 98–105, 107–110, 112, 115, 117–129, 131–138, 140–149, 151]
Interviews	14	[25, 37, 57, 59, 74, 77, 80, 86, 95, 111, 113, 114, 116, 150]
Combination of methods	5	[46, 51, 56, 94, 106]
Email	1	[22]
Unclear/not reported	6	[61, 75, 76, 97, 130, 139]

Mapping starting point	*N*	Citations
Blank page	65	[20, 21, 24, 25, 27, 28, 31, 33, 36, 37, 40, 41, 43, 45, 49, 52, 56, 59, 60, 62, 72, 73, 76, 81, 83, 84, 87, 90, 91, 98, 101, 104–109, 112, 115, 116, 118–128, 131, 134, 136–138, 140–142, 144, 146–150]
Preliminary map (seed model/map)	41	[19, 22, 23, 26, 34, 35, 38, 39, 42, 47, 48, 51, 55, 58, 61, 63, 64, 74, 75, 77, 78, 82, 85, 89, 92, 94, 95, 97, 99, 100, 102, 111, 113, 114, 117, 129, 132, 139, 143, 145, 151]
Unclear/not reported	17	[29, 30, 46, 50, 57, 65, 79, 80, 86, 88, 93, 96, 103, 110, 130, 133, 135]

Location of participatory processes	*N*	Citations
In person	65	[19–21, 23–25, 30, 31, 33, 36, 39, 40, 43, 45, 50, 52, 58, 62–64, 72, 73, 77, 78, 81–84, 87, 90, 91, 93, 98–101, 103, 104, 106–108, 110, 112, 115–121, 124–128, 132, 134, 138, 140, 142, 145–149]
Online	10	[22, 26, 27, 47, 51, 55, 61, 102, 109, 137]
Mixed	9	[41, 79, 105, 114, 122, 123, 135, 136, 141]
Unclear/not reported	39	[28, 29, 34, 35, 37, 38, 42, 46, 48, 49, 56, 57, 59, 60, 65, 74–76, 80, 85, 86, 88, 89, 92, 94–97, 111, 113, 129–131, 133, 139, 143, 144, 150, 151]

Involvement in drawing of maps	*N*	Citations
Participants and researchers/moderators	37	[19, 21, 27, 46, 49, 51, 52, 56, 59, 63, 64, 73, 77, 79, 82, 83, 85, 90, 93, 96, 98, 99, 104, 106, 107, 117, 119–121, 125, 126, 138, 142, 145, 147–149]
Researchers/moderators only	52	[22–26, 31, 35–40, 45, 47, 48, 55, 57, 58, 61, 62, 72, 74, 78, 80, 84, 86, 89, 91, 92, 94, 95, 97, 100–102, 109, 111, 113, 114, 116, 118, 122, 128, 129, 131–133, 139, 140, 144, 146, 150]
Participants only	0	None
Unclear/not reported	34	[20, 28–30, 33, 34, 41–43, 50, 60, 65, 75, 76, 81, 87, 88, 103, 105, 108, 110, 112, 115, 123, 124, 127, 130, 134–137, 141, 143, 151]

Comprehensiveness of reporting on participatory approach	*N*	Citations
Comprehensive reporting	26	[21, 27, 40, 49, 52, 62, 72, 73, 78, 79, 81, 82, 90, 98, 106, 107, 112, 121, 127, 129, 137, 141, 145, 147, 148, 150]
Moderate reporting	62	[19, 20, 23, 25, 26, 28–31, 33, 36–39, 41, 46, 48, 50, 51, 55, 58, 63, 64, 74, 77, 84, 86, 87, 89, 91, 93, 94, 96, 99, 100, 102–105, 108–110, 114, 116, 117, 119, 120, 122–126, 128, 131, 132, 134–136, 140, 142–144]
Limited reporting	29	[22, 24, 34, 35, 42, 45, 47, 56, 57, 59–61, 65, 76, 80, 83, 85, 92, 95, 97, 101, 111, 115, 118, 138, 139, 146, 149, 151]
Very limited reporting	6	[43, 75, 88, 113, 130, 133]

#### Participant profiles

The number of participants involved in mapping processes ranged from 1 to 335. A high proportion of papers did not (clearly) report on sample characteristics (*n* = 43), especially demographic information (e.g. gender, age, role in organisations). The median number of participants involved in mapping was 20. Participants generally represented a multisectoral group (102/123); only 14 studies involved participants from a single sector or discipline, and 7 papers had unclear or no reporting on this aspect. A small number of studies involved intervention beneficiaries, end users or people with lived experiences of the issue under investigation (e.g. [25–31]).

#### Participation in map building and validation stages

Where reported, about half of the studies involved participants in both the map building and map validation stages (*n* = 60), 32 studies in the map building stage only, and 27 in the validation stage only. We found unclear reporting on the stages of stakeholder participation in 23 papers.

Among studies that involved stakeholders in the map building or validation stages, a majority did this solely via workshops (*n* = 97). Some papers explicitly mentioned the use of group model building (GMB), a more formalised yet adaptable approach that includes a collection of workshop scripts emanating from the systems dynamics field [32]. Fewer studies built individual maps during single-person interviews (*n* = 14) (with maps later aggregated by the research team), used a mixed approach (i.e. a combination of workshops, interviews and surveys) (*n* = 5) or sent the maps via email for validation (*n* = 1). Information on primary data collection was unclear in six papers.

A total of 41 studies started map building from a seed model (preliminary map to build on or guide their own map). However, most map building started from a blank page (*n* = 65). This information was unclear or not reported on in 17 studies.

About half of map building processes took place in person (*n* = 65) (e.g. through workshops or interviews), followed by online (*n* = 10) or a mixed approach (both online and in person) (*n* = 9). Overall, 39 studies did not report on this. Where employed, the number of workshops varied from 1 to 49. The median number of workshops was three (21 studies did not report the number clearly). Studies with a high number of workshops tended to use CLD and/or SD methods.

In terms of drawing or refining the maps, we found that, where reported, most of the mapping was carried out by researchers or moderators (*n* = 52), followed by both participants and researchers or moderators (*n* = 37). This information was unclear or not reported in 34 papers. The median number of map iterations (versions of the map) was four, among papers in which this was described. Reporting on this was often unclear, and in 81 papers, absent.

Where reported, participatory systems mapping and/or modelling timeframes varied from half a day [33] to 4 years [19], with several other mapping/modelling processes taking place over a 2-year period [34–37]. Studies that took place over a longer period justified this in terms of the need to build relationships with stakeholders, accommodate different schedules, and adapt to COVID-19 pandemic restrictions. Most of these studies also mapped or modelled wider systems (i.e. national system) and were part of larger projects. Harrison et al. 2023 highlighted the importance of allocating sufficient time for individuals to engage with the system map or model, enabling them to effectively use and share it within their organisations, communities and networks [38]. Many studies (*n* = 71) did not report on these timeframes.

#### Reflexivity and reporting on participatory approach

We also examined whether studies reflected on the participatory approach. Around half the papers (*n* = 69) included a commentary on lessons learnt (i.e. benefits, challenges) relating to the involvement of stakeholders in systems mapping. Most studies commented on the benefits of the process, with few studies discussing challenges. We identified 10 overarching reflexivity themes, which are described in Table 4. In summary, participatory system mapping was valued for fostering collaboration, capturing diverse perspectives and providing a comprehensive understanding of complex systems. Effective engagement depended on careful stakeholder recruitment, sufficient resources and attention to context and feasibility. Structured facilitation and staged processes supported learning, capacity building and inclusion of multiple viewpoints. Studies reported increased knowledge, empowerment and collaboration among participants, while projects benefited from richer insights and, in some cases, system-level change.

**Table 4:** Thematic overview of reflections on participatory approach to systems mapping

Theme	Reflections
**Relevance of participatory approach to systems mapping**	*Identified benefits and other insights:* · Value of cross-disciplinary collaboration in mapping processes · Value of longer-term collaborations and partnerships for systems mapping (e.g. for building shared commitment and trust [137]) · Staged process viewed as facilitating perspective shifts among stakeholders · Group discussions viewed as facilitating learning among stakeholders and building confidence in process · Useful to reflect on and address structural and enduring problems · Useful to gain a comprehensive view on the system and issue · Adaptability of methods allows tailoring of approaches to stakeholders and communities · System maps viewed as a useful visual tool to initiate conversations with stakeholders *Identified challenges*: · Utility of the system map can be short-lived as the system or community changes · Further evaluation of PSM processes are needed to assess effectiveness and relevance
**Resources**	*Identified benefits and other insights:* · Using a seed model (i.e. preliminary map) allows more time for participant involvement · Importance of planning sufficient time for capacity building of participants *Identified challenges*: · Insufficient time for mapping or validation due to limited availability of participants · Significant time commitment for participants · Resource-intensive process for research team
**Cultural or contextual appropriateness**	*Identified benefits and other insights:* · Importance of integration of culture and/or historical perspectives into processes (e.g. following Māori cultural process for building relationships [147])
**Stakeholder recruitment and sampling**	*Identified benefits and other insights:* · Value of investing time for recruitment and building relationships with stakeholders prior to mapping · Importance of engaging stakeholders early on in systems mapping, e.g. in project design stage · Value of involving a wide range of stakeholders · Importance of involving people with lived experience, community members, and organisations or stakeholders on the periphery of a system · Importance of involving decision-makers or community leaders in system of interest to support future actions and implementation *Identified challenges:* · Reflection on how stakeholder sample may have affected final system map (including a reflection on absent stakeholders) · Challenges in recruiting beyond known networks
**Capacity building**	*Identified benefits and other insights:* · Map building viewed as a staged capacity building process · Importance of empowering and building capacity of participants not only in terms of systems methods but also on population health topic (e.g. handing out materials ahead of workshops; improving map visuals to support engagement in workshops [79]) · The need to support relationship- and network-building across sectors or groups *Identified challenges:* · The difficulty to convey complexity of system in accessible ways across a range of audiences (e.g. to mitigate this, one project developed a summary report for community audiences [31])
**Facilitation**	*Identified benefits and other insights:* · Importance of acknowledging researcher positionality within process · Importance of good and consistent facilitation throughout mapping processes (e.g. to balance power dynamics [118]; to maintain consistency of processes between sessions [29]) · Importance of accounting for discordant views and negotiating consensus among participants · Usefulness of using Scriptapedia (Hovmand et al., 2012) for ease of engagement with stakeholders
**Group dynamics and participant engagement**	*Identified benefits and other insights:* · Importance of exposure to and engagement with discordant views among participants to support the generation of new insights and meaning · Smaller groups perceived to improve participation and debate · Usefulness of acknowledging and discussing challenges in mapping process with participants · Usefulness of collecting workshop feedback to improve participant engagement *Identified challenges:* · Uncertainty about whether it was feasible for all stakeholders to engage equally in systems mapping processes · How to treat discordant or minority views in the final system map (e.g. Savona et al., 2021)
**Feasibility of stakeholder involvement**	*Identified benefits and other insights:* · Importance of taking into account barriers to involvement (e.g. childcare, location of workshops [121]) · Enabling participation in stakeholders’ own time to support engagement · Closely timed workshops viewed as enhancing engagement and group reflection from one session to the next · Online processes enable a wider group of stakeholders to take part or to uphold anonymity of participants (e.g. people with lived experience [26]; competing organizations or companies [150]) · Designing shorter map building sessions to increase engagement · Inclusion of financial compensation for participation
**Outcomes (for participants)**	*Identified benefits and other insights:* · Knowledge sharing · Perspective shifts (i.e. change in mental models) · A sense of empowerment among participants · Sense of ownership of model by participants · Trust and confidence in process and final output · Identification of shared interests and values · Identification of location of resources in the system · Development of a common agenda and vision · A more comprehensive and nuanced understanding of the issue or system of interest · Enhanced critical thinking and solutions-focussed thinking among participants · Participants’ recognition of their influence on the system · Stakeholders seeing themselves as part of the solution · Increased collaboration across sectors or disciplines · Networking among stakeholders · Collaboration in systems mapping seen to increase likelihood of future collaboration on systems change
**Outcomes (for project or system of interest)**	*Identified benefits and other insights:* · Process enabled the identification of factors that had not been previously identified in studies or reviews · Researchers gained an understanding of stakeholders’ values · Process led to systems change (e.g. context-adapted policy formulation [62]; changes in service delivery [112]) · System mapping outputs seen as useful for research engagement beyond study (e.g. enabling decision-makers to ask *what if* questions and discuss different courses of action [133])

We also assessed the comprehensiveness of reporting on the participatory approach. The assessment was based on eight criteria developed by the authors on the basis of their knowledge of PSM processes (Box 1). These criteria covered key elements such as participation scope and context, depth and quality of involvement, and clarity of reporting and reflection. We found that only 26/123 papers provided a comprehensive reporting of the participatory approach (fulfilled 7–8 criteria), followed by 62 papers with moderate reporting (5–6 criteria), 29 papers with limited reporting (3–4 criteria) and six with very limited reporting (1–2 criteria). Most notably criteria 6, 7 and 8 were key areas where reporting was more limited (see citations under Table 3).

**Box 1:** Assessment criteria on reporting of participatory approach

Assessment criteria on reporting of participatory approach
1. Stages of participation (i.e. at which stages of the study were participants involved?) 2. Type of participation (i.e. how were participants involved [e.g. workshops, interviews]?) 3. Sample and participant profiles (i.e. how many participants took part, and what were their sociodemographic profiles?) 4. Interdisciplinarity (i.e. what were the professional or disciplinary backgrounds of participants?) 5. Level of participation (i.e. how much were participants involved?) 6. Description of processes (i.e. does the study clearly outline how participants were engaged, including key steps and activities?) 7. Clarity of overall reporting on participatory processes (i.e. is the reporting of processes easy to identify and understand?) 8. Reflection on the participatory approach (i.e. does the paper present a reflection on the participatory approach?)

Finally, we found that very few studies included a formal evaluation of stakeholders’ views on their involvement in the mapping process (*n* = 4/123), for example, through surveys and interviews. Feedback was provided during or immediately after workshops [39, 40] or collected months later [28, 41]. However, it is possible that some studies may have reported evaluation data elsewhere.

### Map features and properties

#### Secondary data sources

In addition to the use of primary data for map building and validation (e.g. mapping workshops, interviews), a significant number of studies also used secondary data (*n* = 52). This consisted of literature reviews (*n* = 30), document analysis (*n* = 12), interview data (*n* = 12), routine/surveillance data (*n* = 12), group discussion data (*n* = 6) and survey data (*n* = 5). One study included intervention evaluation data [42] (Table 5).

**Table 5:** Map features and properties

Secondary data sources	*N*	Citations
Literature reviews	30	[22, 27, 35, 38, 39, 43, 47, 48, 55, 58, 72–74, 77–79, 82, 92, 95, 97, 102, 111, 117, 120, 129, 130, 132, 141, 143, 145]
Document analysis	12	[30, 35, 51, 62, 65, 77, 92–94, 99, 100, 139]
Interview data	12	[20, 45, 63, 64, 77, 86, 93, 97, 113, 116, 139, 145]
Group discussion data	6	[38, 39, 45, 60, 93, 138]
Survey data	5	[30, 75, 95, 117, 132]
Routine/surveillance data	12	[25, 30, 42, 43, 56, 65, 77, 95, 97, 112, 129, 130]

Map properties	*N*	Citations
Direction of influence	123	[19–31, 33–43, 45–52, 55–65, 72–151]
Nature of connection (polarity)	98	[19–24, 26–29, 31, 33, 34, 38, 40, 41, 43, 45–49, 55–61, 63, 64, 72–74, 76, 78, 80–82, 85–89, 91–94, 96, 97, 99, 100, 102–110, 112–129, 132–149, 151]
Delays	21	[21, 23, 28, 31, 38, 45, 46, 57, 61, 84–86, 105, 107, 108, 116, 120, 129, 131, 137, 138]
Strength of connection	9	[29, 51, 59, 78, 102, 125, 133, 144, 146]
Feedback loops	69	[19, 20, 22–24, 28, 31, 34, 35, 37, 38, 40, 41, 43, 45, 46, 52, 56–58, 63, 64, 72, 73, 76, 77, 80–86, 88, 89, 92, 93, 96, 97, 99, 102, 105, 108, 112, 113, 115, 116, 119, 121, 122, 126, 128–130, 132, 134–143, 145, 147–149]
Conditional probabilities	2	[75, 111]
Clusters/groupings	57	[19, 23–27, 35, 40, 41, 46–52, 55, 60–64, 72, 78, 81–84, 91, 99, 101, 103, 105–107, 109–111, 113–115, 118–121, 123, 127, 135–139, 145, 147, 148, 150, 151]

#### Map properties

Overall, we found limited labelling and analyses of maps. Labelling included the direction of influence (*n* = 123), nature of connection (polarity) (*n* = 98), delays (*n* = 21), strength of influence (*n* = 9) and conditional probabilities (*n* = 2). See Additional File 4 for definitions of terms. In about half the papers, subsections of the maps were organised in thematic clusters (*n* = 57). Many maps included labelled feedback loops (*n* = 69), reflecting the predominance of CLDs in the sample. One paper incorporated labels to illustrate and describe the feedback loop themes [21], another added dotted lines to feedback loops where discrepancies existed between those identified by stakeholders and the literature versus those indicated by local data [43] (Table 5).

### Integration of other conceptual frameworks and methods

Finally, we looked at whether studies had integrated methods and conceptual frameworks other than those directly related to PSM approaches. We found that only a minority of papers (*n* = 24) integrated other methods and/or conceptual frameworks. Methods more directly related to systems thinking included: viable systems model (VSM) [36], systemic intervention methodology [44–46], systems network analysis [36, 47–50] and cybernetic foundations and engineering systems design [51]. Other methods and frameworks included integration of the mãtauranga Mãori worldview [52] (for further reading on Indigenous knowledge and systems, see McKelvie-Sebileau et al. [53] and Goodchild [54]), theories of change [55–57], socioecological framework [58, 59], realist approaches [34, 55, 60–62] and grounded theory [63–65].

## Discussion

This systematic scoping review is the first to examine the use of a range of PSM approaches in the population health field. It enables reflection on both past applications of PSM methods and on future directions in the field. Overall, we found a growing trend in the use of these methods in population health research, with 58/123 studies meeting our inclusion criteria published since 2020. We found considerable variability in study design, application and reporting of these methods. We also observed innovative approaches to study design, further shaping the evolving landscape of these methods and presenting opportunities for their development. Geographically, these methods were most widely used in Australia and the United States, with a notable increase in geographical diversity since 2021.

### Considerations on method choice and design

Regarding choice of method and study design, most studies employed approaches grounded in systems dynamics [66], either formally or through adaptations (whether explicitly or implicitly), with a notable focus on using qualitative causal loop diagrams (CLDs) as a stand-alone method. Similar to van der Akker et al. (2023), we found significant variation in the way studies were designed. Flexibility in design is considered inherent to this field, as the systems under study and the purpose of the mapping can vary significantly [3, 4]. However, we suggest that this openness may also indicate a need for more comprehensive methodological guidance and training and more cross-learning between disciplines using these methods. A bibliometric analysis looking at model validation literature found different validation practices and minimal citation across fields (i.e. environmental sciences, economic sciences); this could be better harnessed in the future [67].

We observed minimal integration of conceptual frameworks and methods beyond this field. In their review, Baugh Littlejohns et al. also found limited reporting on the methodological underpinnings of causal loop diagrams [7]. Our review also found that most studies aimed to refine understanding of issues or contexts, as opposed to a focus on more applied objectives such as intervention or policy development or refinement [7]. We speculate that this could be due to the relatively new adoption of these methods in population health research or to a lack of methods development in this area. Interestingly, Hovmand et al. suggested that mapping, particularly through group model building (GMB), can be understood as an intervention, as its success or failure can have significant implications for future stakeholder collaboration on complex issues [68]. Recent guidance developed by the authors of this current paper responds to the identified variability in design and method reporting. It includes a Design Framework to guide the choice and design of PSM. This framework aims to broaden the perspective on the applications of systems mapping, clarifies its purposes and informs the design of participatory approaches [3].

### Considerations on design of participatory processes

Our findings demonstrated that, to date, participatory approaches have been more frequently employed for qualitative system maps compared with quantitative system maps, although we noted a recent increase in the use of participatory methods for quantitative mapping methods (especially SD modelling). Notably, 27 studies in our sample did not involve participants in the map validation process. This finding, consistent with observations by van den Akker et al. [8], is somewhat surprising, as validation is recommended as a crucial final step in ensuring that a system map accurately reflects participants’ views.

While multi-stakeholder involvement was common, the inclusion of participants with lived experience was limited, which may reflect the methodological demands of PSM approaches – and the additional time and capacity required to meaningfully engage a broader and more diverse group of stakeholders. Consideration of other factors such as gender and age of participants were often unclear. Evaluation of participatory processes (e.g. post-workshop surveys or interviews) was limited, a finding also identified by van den Akker et al. [8]. Many papers, however, included reflections on stakeholder participation, drawing either from researchers’ perspectives on the process or insights captured in study field notes. Finally, there is likely a distinction between engaging stakeholders in qualitative mapping versus quantitative modelling. Consequently, the capacity-building requirements of these approaches differ substantially [38]. Box 2 presents a synthesis of key lessons learnt on involving stakeholders in systems mapping (details are presented in Table 4).

**Box 2:** Further synthesis of lessons learnt on involving stakeholders in systems mapping

**Cross-disciplinary and inclusive collaboration**
Participatory systems mapping requires collaboration across sectors, disciplines and stakeholder groups, including individuals with lived experience. Engaging decision-makers and community leaders enhances implementation potential, while recruitment challenges highlight the need for proactive relationship-building beyond established networks
**Capacity building and engagement**
Participatory systems mapping is enhanced through staged capacity building processes that equip participants with technical skills and issue-specific knowledge. Smaller, consistently facilitated groups promote meaningful participation and debate, while flexible engagement options, such as online access and childcare support, foster inclusivity
**Mapping as a tool for learning, perspective shifts and shared ownership**
Participatory systems mapping facilitates learning, critical thinking and shifts in mental models through group discussions and exposure to diverse perspectives. This process fosters shared ownership, enhances stakeholder engagement and builds trust by encouraging participants to see themselves as part of the solution
**Adaptability and reflection**
Participatory systems mapping requires adaptability to accommodate diverse stakeholders and contexts, while ongoing reflection on challenges and missing perspectives strengthens accuracy and relevance. Evaluation and participant feedback supports further refinement and improvement of future mapping processes
**Long-term value and impact**
Sustained partnerships and collaborations enhance the long-term effectiveness of systems mapping (e.g. fostering policy change, service improvements and cross-sector cooperation). However, as systems evolve, regular updates and assessments are essential to maintain the maps’ relevance and impact

### Considerations on reporting

Regarding the breadth and quality of reporting, there was a notable lack of detail in reporting of study methods, particularly concerning study procedures (how the map was built and validated) and stakeholder involvement. We acknowledge the challenge of balancing the clear presentation of complex methods and data with the strict word limits imposed by some journals; however, supplementary files could be better utilised to provide additional detail. Insufficient reporting poses challenges for reproducibility of methods and in turn slows methodological development in an already quite ill-defined set of approaches [4]. The GMB workshop scripts from the systems dynamics field (e.g. via open repositories, such as Scriptapedia) provide valuable insights and experience [68]. However, there is a need to further develop such scripts into more detailed methods, which can also serve as a guide for researchers to report on study procedures more comprehensively.

Many of the methods identified in the review (e.g. systems dynamics, fuzzy cognitive mapping and Bayesian belief networks) originate from engineering and mathematics fields, which traditionally use less participatory approaches or qualitative research methods than the population health research field. Consequently, the application of PSM methods in population health contexts could be at an opportune moment of methods development with potential for further integrating social and behavioural science theories and methods, qualitative research principles and reporting guidelines (e.g. [69]), as well as involvement frameworks such as the National Institute for Health and Care Research (NIHR) United Kingdom standards for public involvement [70] and the NIHR School for Public Health Research (SPHR) public involvement resources [71]. The assessment criteria presented in Box 1 provide a useful starting point and framework that future researchers can use to improve the design and reporting of participatory approaches in systems mapping studies.

In their review, Baugh Littlejohns et al. also suggested further integration with action research and community-based participatory research approaches [7]. While the complexity of mapping processes may partly account for limited reporting, a subset of studies demonstrated enhanced methodological transparency.

Only a few studies provided critical reflections or limitations related to the use of participatory approaches (e.g. challenges in recruitment or group dynamics). Finally, a small number of studies integrated or reflected on the theoretical frameworks underlying the use of these methods. This omission not only diminishes the effectiveness of systems mapping research in guiding policy development and designing population health interventions but also represents a missed opportunity in further developing these methods within social sciences and population health, as well as developing their explanatory power in examining their subject matter.

### Strengths and limitations

Our scoping review presents several strengths that enhance the rigour and comprehensiveness of its findings. This includes the involvement of an information specialist in the search design, the use of independent double-screening of titles and abstracts and at the full-text screening stage, and the inclusion of additional papers through citation tracking, where we extracted relevant studies from systematic reviews and protocols. This ensured that studies not directly captured in our initial search were also considered. In terms of limitations, the use of a limited number of databases and a search restricted to academic publications (excluding grey literature) may have led to the omission of relevant studies, affecting the review’s comprehensiveness. Although our search aimed to capture a wide range of causal systems mapping methods, including concept maps, some relevant studies may have been missed. By excluding *closed* systems, our findings apply mainly to the use of participatory systems mapping in open, population-level contexts and may not directly reflect studies focussing on tightly bound or organisational systems. We also extracted data solely on stakeholder participation in map building and validation, possibly omitting data on their involvement in map analysis, use and dissemination. Consequently, while our review effectively maps existing evidence in population health, it may not encompass the full breadth of available research.

## Conclusions and future directions

The use of PSM in population health remains a work in progress, with growing application and ongoing innovation. Further development and refinement are needed to advance these methods effectively. While some flexibility is inherent to these methods, it is also necessary to explore which aspects could be further standardised. To advance the field of participatory systems mapping, four key steps are proposed:

- Advance methodological development: We identified substantial variation in study design and a limited use of theoretical or conceptual frameworks to guide methodological choices and interpretation, highlighting an important area for further development in PSM. To address this, future work should more deliberately integrate theory and method, document experiences across different approaches and develop clearer methodological guidance to enhance consistency and transparency.
- Strengthen participatory approaches: Our review shows that participation in map building and validation stages was uneven, and that involvement of individuals with lived experience was limited. Future work should therefore emphasise participatory approaches that meaningfully engage diverse stakeholders throughout the mapping cycle and develop strategies to manage the resource and capacity demands of inclusive engagement.
- Improve reporting and reflexive transparency: Given the frequent lack of detail in reporting study methods and limited reflexivity on research processes, clearer reporting standards are essential. Developing reporting guidelines for PSM could enhance transparency, support reproducibility and accelerate methodological learning within the field.
- Develop skills and communities of practice: The variability we observed in study design possibly reflects uneven access to knowledge and skills in this field. To support high-quality participatory systems mapping, future work should prioritise capacity-building initiatives, practical guidance materials (e.g. [3, 4, 32]) and communities of practice that bridge population health with other fields also using and developing these methods.

Taken together, these steps provide a pathway for strengthening the quality, consistency and impact of participatory systems mapping in population health, and can be enacted through coordinated efforts in methodological guidance, reporting standards, capacity building and cross-disciplinary collaboration.

## Supplementary Information

Supplementary Material 1.

Supplementary Material 2.

Supplementary Material 3.

Supplementary Material 4.

## Data Availability

The dataset supporting the conclusions of this article is included within the article (and its additional file(s)).

## Abbreviations

BBN: Bayesian belief networks
CECAN: Centre for the Evaluation of Complexity across the Nexus
CLD: Causal loop diagram
FCM: Fuzzy cognitive mapping
GMB: Group model building
NIHR: National Institute for Health and Care Research
OECD: Organisation for Economic Co-operation and Development
PRISMA-ScR: PRISMA extension for scoping reviews
PSM: Participatory systems mapping
SD: Systems dynamics
SPHR: School for Public Health Research
VSM: Viable systems model
