# Supplementary material for: Participatory systems mapping: a review of population health research practice
Source: Health Res Policy Syst. 2026 Mar 10;24:30. doi: 10.1186/s12961-026-01457-6 (PMC13047807; doi:10.1186/s12961-026-01457-6)
Supplement: Supplementary file 1 — Supplementary Material 1. [file 12961_2026_1457_MOESM1_ESM.docx]

**Additional file 1_Search strategies**

*Search strategy for Ovid MEDLINE(R)*

| **#** | **Searches** |
| --- | --- |
| 1 | "Social Determinants of Health"/ |
| 2 | Delivery of Health care/ or Health Status/ or Population Health/ or Health Status Indicators/ or Public policy/ or Population characteristics/ |
| 3 | (population adj1 health).ab,kf,ti. |
| 4 | (health adj1 (policy or determinants or inequalities or behavio?r or status)).ab,kf,ti. |
| 5 | social determinants of health.ab,kf,ti. |
| 6 | Public Health/ |
| 7 | Health Education/ |
| 8 | Public Health Administration/ |
| 9 | Public Health Practice/ |
| 10 | health planning/ |
| 11 | health promotion/ |
| 12 | health behavior/ |
| 13 | Demography/ |
| 14 | (pandemic* or epidemic*).ab,ti. |
| 15 | Epidemics/ or Pandemics/ |
| 16 | 1 or 2 or 3 or 4 or 5 or 6 or 7 or 8 or 9 or 10 or 11 or 12 or 13 or 14 or 15 |
| 17 | (dynamic adj1 model?ing).ab,ti. |
| 18 | "“System* map*”".ab,kf,ti. |
| 19 | (system* adj1 thinking).ab,kf,ti. |
| 20 | (system adj1 model?ing).ab,kf,ti. |
| 21 | "causal loop diagram*".ab,kf,ti. |
| 22 | "Fuzzy cognitive map*".ab,kf,ti. |
| 23 | "Bayesian Belief Network*".ab,kf,ti. |
| 24 | Bayesian network*.ab,kf,ti. |
| 25 | "System* Dynamic*”".ab,kf,ti. |
| 26 | "cognitive map*".ab,kf,ti. |
| 27 | complex adaptive system*.ab,kf,ti. |
| 28 | (complexity adj1 theor*).ab,kf,ti. |
| 29 | (complexity adj1 science).ab,kf,ti. |
| 30 | Causal map.ab,kf,ti. |
| 31 | causal loop.ab,kf,ti. |
| 32 | Causal diagram*.ab,kf,ti. |
| 33 | Causal model*.ab,kf,ti. |
| 34 | Causal linkage*.ab,kf,ti. |
| 35 | "participatory map*".ab,kf,ti. |
| 36 | participatory model*.ab,kf,ti. |
| 37 | (theory adj1 change).ab,kf,ti. |
| 38 | 17 or 18 or 19 or 20 or 21 or 22 or 23 or 24 or 25 or 26 or 27 or 28 or 29 or 30 or 31 or 32 or 33 or 34 or 35 or 36 or 37 |
| 39 | 16 and 38 |
| 40 | exp animals/ not humans.sh. |
| 41 | 39 not 40 |
| 42 | limit 41 to yr="2000 -Current" |

*Search strategy for Scopus*

( ( TITLE-ABS-KEY ( pandemic* OR epidemic* OR "social determinants of health" ) ) OR ( TITLE-ABS-KEY ( health W/1 ( planning OR promotion OR policy OR determinants OR inequalities OR behavio?r OR status ) ) ) OR ( TITLE-ABS-KEY ( population W/1 health ) ) ) AND ( ( TITLE-ABS-KEY ( "system* mapping" OR "dynamic model?ing" OR "system* W/1 thinking" OR "system W/1 model?ing" OR "causal loop diagram*" OR "Fuzzy cognitive map*" ) ) OR ( TITLE-ABS-KEY ( "Bayesian Belief Network*" OR "Bayesian network*" OR "System* Dynamic*" OR "cognitive map*" OR "complex adaptive system*" OR "complexity W/1 theor*" OR "complexity W/1 science" ) ) OR ( TITLE-ABS-KEY ( "Causal map" OR "causal loop" OR "Causal diagram*" OR "Causal model*" OR "Causal linkage*" OR "participatory map*" OR "participatory model*" OR "theory W/1 change" ) ) ) AND PUBYEAR > 2020 AND PUBYEAR < 2024 AND ( EXCLUDE ( SUBJAREA , "ENGI" ) OR EXCLUDE ( SUBJAREA , "BIOC" ) OR EXCLUDE ( SUBJAREA , "MATH" ) OR EXCLUDE ( SUBJAREA , "AGRI" ) OR EXCLUDE ( SUBJAREA , "PHAR" ) OR EXCLUDE ( SUBJAREA , "PHYS" ) OR EXCLUDE ( SUBJAREA , "IMMU" ) OR EXCLUDE ( SUBJAREA , "DENT" ) OR EXCLUDE ( SUBJAREA , "EART" ) OR EXCLUDE ( SUBJAREA , "CENG" ) OR EXCLUDE ( SUBJAREA , "MATE" ) OR EXCLUDE ( SUBJAREA , "ENER" ) OR EXCLUDE ( SUBJAREA , "CHEM" ) OR EXCLUDE ( SUBJAREA , "VETE" ) )
