# Supplementary material for: Participatory systems mapping: a review of population health research practice
Source: Health Res Policy Syst. 2026 Mar 10;24:30. doi: 10.1186/s12961-026-01457-6 (PMC13047807; doi:10.1186/s12961-026-01457-6)
Supplement: Supplementary file 2 — Supplementary Material 2. [file 12961_2026_1457_MOESM2_ESM.docx]

**Additional file 2. Data extraction categories**

| *EXTRACTION FIELDS* | *RESPONSE CATEGORIES* |
| --- | --- |
| **Paper identification** |  |
| Author(s) | *[open text]* |
| Year | *[open text]* |
| Link to online version | *[open text]* |
| **General design** |  |
| Type of PSM method(s) | - *Bayesian belief network* - *Fuzzy cognitive map* - *Causal loop diagram* - *Stock and flow diagram* - *System dynamics model* - *Systems-based theory of change* - *CECAN PSM* - *Other (please specify)* |
| Stated purpose of mapping | *[open text]* |
| Type of study | - *Stand-alone research* - *Part of a wider project* - *Unclear or not reported* |
| **Context & topic area** |  |
| Country | *[open text]* |
| Geographical/spatial context | - *Local/localised* - *Sub-national region (e.g. North of Scotland)* - *National* - *Supranational region (e.g. Europe)* - *Global* - *Unclear or not reported* |
| Context stability | - *Stable context* - *Fragile context* - *Unclear or not reported* |
| Disturbances in stable contexts | *[open text]* |
| Hypothetical/specific context | - *Specific case study / spatially located* - *Abstract context / generalised context* - *Unclear or not reported* |
| Population health area(s) | *[open text]* |
| **Participatory approach** |  |
| Stage(s) of participation | - *Map building* - *Map validation* - *Other (please specify)* - *Unclear or not reported* |
| Type of participation | - *Group model/map building* - *Individual map building (e.g. interviews)* - *Unclear or not reported* |
| Inter-disciplinarity of group involved in mapping | - *Multi sector/discipline* - *Single sector* - *Unclear or not reported* |
| Number of workshops | *[open text]* |
| Number of participants | *[open text]* |
| Is there a reflection on the participatory approach? | - *Yes* - *No* |
| Scope of reporting on participation | - *Stages of participation (i.e. at which stages of the study were participants involved?)* - *Type of participation (i.e. how were participants involved (e.g. workshops, interviews)?)* - *Sample and participant profiles (i.e. how many participants took part, and what were their socio-demographic profiles?)* - *Inter-disciplinarity (i.e. what were the professional or disciplinary backgrounds of participants?)* - *Level of participation (i.e. how much were participants involved?)* - *Description of processes (i.e. whether the study clearly outlines how participants were engaged, including key steps and activities?)* - *Clarity of overall reporting on participatory processes (i.e. is the reporting of processes easy to identify and understand?)* - *Reflection on the participatory approach (i.e. does the paper present a reflection on the participatory approach?)* |
| Platform/space | - *In person* - *Online* - *Mixed* - *Unclear or not reported* |
| Map/model creators (i.e. who drew the maps?) | - *Participant(s)* - *Moderators/researchers* - *Both* - *Unclear or not reported* |
| Timeline | *[open text]* |
| Number of map/model iterations | *[open text]* |
| Were map or elements pre-constructed or based on other data prior to participatory processes? | - *Yes* - *No* - *Unclear or not reported* |
| **Map features and properties** |  |
| Use of secondary data | - *Interview data* - *Group discussion data* - *Survey data* - *Routine data/surveillance data* - *Literature review* - *Document analysis (e.g. policy analysis)* - *Other data (to specify)* - *Unclear or not reported* |
| Map properties | - *Direction of influence* - *Nature of connection (i.e. polarity)* - *Feedback loops* - *Strength/degree of connection* - *Delays* - *Conditional probabilities* - *Clustering/grouping (e.g. colour-coding)* - *Other (please specify)* |
| Other methods and frameworks | *[open text]* |
| Authors’ reflections on participatory approach to systems mapping | *[open text]* |
| Additional information | *[open text]* |
