# Supplementary material for: Participatory systems mapping: a review of population health research practice
Source: Health Res Policy Syst. 2026 Mar 10;24:30. doi: 10.1186/s12961-026-01457-6 (PMC13047807; doi:10.1186/s12961-026-01457-6)
Supplement: Supplementary file 3 — Supplementary Material 3. [file 12961_2026_1457_MOESM3_ESM.docx]

**Additional file 3. Study characteristics**

| **Ref #** | **Author(s)/year** | **Country(ies)** | **Method(s)** |
| --- | --- | --- | --- |
| 19 | Brennan et al. 2015 | USA, Puerto Rico | CLD |
| 20 | Guariguata et al. 2021 | Barbados, Belize, Jamaica, and Saint Vincent and the Grenadines | CLD |
| 21 | Langellier et al. 2019 | Peru, Brazil, Guatemala | CLD |
| 22 | Ozawa et al. 2016 | Low and middle income countries | CLD |
| 23 | Pineo et al. 2020 | Australia and USA | CLD |
| 24 | Savona et al. 2021 | UK, Portugal, Poland, Netherlands, and Norway | CLD |
| 25 | Adams et al. 2020 | USA | Other causal map |
| 26 | Bates et al. 2022 | UK | CLD |
| 27 | Chavez-Ugalde et al. 2022 | UK | CLD |
| 28 | Frerichs et al. 2016 | USA | CLD |
| 29 | Giles et al. 2008 | Canada | FCM |
| 30 | Occhipinti et al. 2021 | Australia | S&F; SD |
| 31 | Freedman et al. 2022 | USA | CLD; S&F; SD |
| 33 | Moreland 2015 | USA | CLD |
| 34 | Brown et al. 2022 | Australia | S&F; ToC |
| 35 | Nielsen and Wakeland 2014 | USA | CLD; S&F; SD |
| 36 | Rosas and Knight 2019 | USA | Other causal map |
| 37 | Wakeland et al. 2015 | USA | S&F; SD |
| 38 | Harrison et al. 2023 | New Zealand | CLD |
| 39 | Olabisi et al. 2012 | USA | S&F; SD |
| 40 | Weeks et al. 2017 | USA | CLD |
| 41 | Calancie et al. 2023 | USA | CLD |
| 42 | Kenealy et al. 2012 | New Zealand | S&F; SD |
| 43 | Macmillan et al. 2014 | New Zealand | CLD; SD |
| 45 | Sharma et al. 2023 | Nepal | CLD |
| 46 | Sharma et al. 2021 | Nepal | CLD |
| 47 | Bauer et al. 2023 | USA | CLD |
| 48 | Darwich et al. 2023 | Sweden | CLD |
| 49 | Fergus et al. 2022 | Uganda | CECAN PSM |
| 50 | McGlashan et al. 2016 | Australia | CLD |
| 51 | Hassannezhad et al. 2023 | UK | FCM |
| 52 | McKelvie-Sebileau et al. 2022 | New Zealand | CLD |
| 55 | Allender et al. 2023 | Christmas and Cocos Islands (Australian Indian Ocean Territories) | CLD |
| 56 | Suriyawongpaisal et al. 2021 | Thailand | CLD; ToC |
| 57 | Suriyawongpaisal et al. 2020 | Thailand | CLD |
| 58 | Gillen et al. 2014 | USA | CLD; S&F; SD |
| 59 | Gordon et al. 2020 | USA | FCM |
| 60 | Brown et al. 2019 | Australia | CLD |
| 61 | Koorts et al. 2021 | Australia | CLD |
| 62 | Marchal et al. 2021 | Georgia | CLD |
| 63 | Afdhal et al. 2022 | Indonesia | Other causal map |
| 64 | Bakhtawar et al. 2022 | India | Other causal map |
| 65 | Zhang et al. 2022 | China | S&F; SD |
| 72 | Uleman et al. 2021 | Netherlands | CLD |
| 73 | Reumers et al. 2022 | Netherlands | CLD |
| 74 | Sluijs et al. 2021 | Netherlands | S&F; SD |
| 75 | Ordovas et al. 2023 | Spain | BBN |
| 76 | Desai et al. 2008 | UK | CLD; S&F; SD |
| 77 | Lebcir et al. 2010 | Russia | CLD; S&F; SD |
| 78 | Cavill et al. 2020 | UK | CLD |
| 79 | Stansfield et al. 2021 | UK | Other causal map |
| 80 | Rwashana et al. 2009 | Uganda | CLD |
| 81 | Hendricks et al. 2022 | South Africa | CLD |
| 82 | Lembani et al. 2018 | South Africa | CLD |
| 83 | Osman et al. 2021 | South Africa | CLD |
| 84 | Dianati et al. 2019 | Kenya | CLD; S&F; SD |
| 85 | Semwanga et al. 2016 | Uganda | CLD; SD |
| 86 | Rwashana et al. 2014 | Uganda | CLD |
| 87 | Haroz et al. 2021 | Thailand | CLD; S&F |
| 88 | Hsiao et al. 2023 | Taiwan | CLD |
| 89 | Cheng et al. 2023 | Malaysia | CLD |
| 90 | Urwannachotima et al. 2019 | Thailand | CLD |
| 91 | Zablith et al. 2021 | Lebanon | CLD |
| 92 | Harpring et al. 2021 | Yemen | CLD |
| 93 | Kunpeuk et al. 2020 | Thailand | CLD; S&F |
| 94 | Ameli et al. 2023 | Iran | FCM |
| 95 | Chuong et al. 2021 | China | Other causal map; S&F; SD |
| 96 | Trani et al. 2016 | Afghanistan | CLD; SD |
| 97 | Ahmad et al. 2019 | Pakistan | CLD; S&F; SD |
| 98 | Urwannachotima et al. 2020 | Thailand | S&F; SD |
| 99 | Jamal et al. 2019 | Syria | CLD |
| 100 | Parmar et al. 2021 | Jordan | CLD |
| 101 | Millar et al. 2023 | Australia | CLD |
| 102 | Ali et al. 2024 | Australia | CLD |
| 103 | Brown et al. 2022 | Australia | CLD |
| 104 | Bolton et al. 2022 | Australia | CLD |
| 105 | Sahin et al. 2020 | Australia | CLD |
| 106 | Friel et al. 2017 | Australia | CLD |
| 107 | Allender et al. 2015 | Australia | CLD |
| 108 | Waqa et al. 2017 | Fiji | CLD |
| 109 | Whelan et al. 2021 | Australia | CLD |
| 110 | Roberts et al. 2019 | Australia | Other causal map; S&F; SD |
| 111 | Ho et al. 2016 | Australia | BBN |
| 112 | Brown et al. 2022 | Australia | S&F; SD |
| 113 | Boelsen-Robinson et al. 2021 | Australia | CLD |
| 114 | Koorts et al. 2022 | Australia | CLD |
| 115 | Maitland et al. 2021 | Australia | CLD |
| 116 | Owen et al. 2018 | Australia | CLD |
| 117 | Skouteris et al. 2015 | Australia | CLD; SD |
| 118 | Deutsch et al. 2022 | USA | CLD |
| 119 | Trani et al. 2024 | USA | CLD |
| 120 | Payne-Sturges et al. 2023 | USA | CLD |
| 121 | Poon et al. 2022 | Canada | CLD |
| 122 | Hosseinichimeh et al. 2022 | USA | S&F |
| 123 | Calancie et al. 2022 | USA | CLD |
| 124 | Nelson et al. 2015 | USA | CLD |
| 125 | Giles et al. 2007 | Canada | FCM |
| 126 | Thomas and Reilly 2015 | USA | CLD |
| 127 | Keane et al. 2015 | USA | CLD |
| 128 | Marcal et al. 2021 | USA | CLD |
| 129 | Weeks et al. 2020 | USA | CLD; S&F; SD |
| 130 | Wakeland et al. 2013 | USA | S&F; SD |
| 131 | Bridgewater et al. 2011 | USA | Other causal map; S&F; SD |
| 132 | Batchelder et al. 2015 | USA | CLD; SD |
| 133 | Mahamoud et al. 2013 | Canada | CLD; SD |
| 134 | Kok et al. 2015 | Canada | CLD; S&F; SD |
| 135 | Hassmiller Lich et al. 2017 | USA | CLD; S&F; SD |
| 136 | Egbuonye et al. 2022 | USA | CLD |
| 137 | Gullett et al. 2022 | USA | CLD |
| 138 | Mui et al. 2019 | USA | CLD |
| 139 | Sabounchi et al. 2023 | USA | CLD |
| 140 | BeLue et al. 2012 | USA | CLD |
| 141 | Pronk et al. 2023 | USA | CLD |
| 142 | Matson et al. 2022 | USA | CLD |
| 143 | Smith et al. 2021 | Unclear | CLD |
| 144 | Weimer-Jehle et al. 2012 | Unclear | Other causal map |
| 145 | Moustaid et al. 2020 | Unclear | CLD; S&F; SD |
| 146 | Titz and Doll 2009 | Unclear | Other causal map |
| 147 | Gerritsen et al. 2020 | New Zealand | CLD |
| 148 | Gerritsen et al. 2019b | New Zealand | CLD |
| 149 | Cavana and Clifford 2006 | New Zealand | CLD; S&F; SD |
| 150 | Gerritsen et al. 2019a | New Zealand | Other causal map |
| 151 | Signal et al. 2013 | New Zealand | CLD |

*Abbreviations:* BBN = Bayesian Belief Network, CLD = Causal Loop Diagram, FCM = Fuzzy Cognitive Map, S&F = Stock and Flow diagram, SD = Systems Dynamics model.
