# Supplementary material for: Participatory systems mapping: a review of population health research practice
Source: Health Res Policy Syst. 2026 Mar 10;24:30. doi: 10.1186/s12961-026-01457-6 (PMC13047807; doi:10.1186/s12961-026-01457-6)
Supplement: Supplementary file 4 — Supplementary Material 4. [file 12961_2026_1457_MOESM4_ESM.docx]

**Additional file 4. Definitions of methods and concepts**

**Definitions of methods***

| Systems based Theory of Change (ToC) maps | ‘Systems-based ToC mapping is an emerging method that is designed to overcome challenges of established ToC models (e.g. typically linear models). [...] Systems-based ToC mapping removes the need to assume linear, acyclic change by establishing the complex causal arrangement of factors between points of intervention and outcomes. [...] This approach is distinct from, but related to, methods such as programme theory development, logic mapping, logic modelling, results chaining, or outcome mapping’. |
| --- | --- |
| Causal Loop Diagrams (CLD) | ‘Causal loop diagrams provide a qualitative picture of a system’s structure. One of their purposes is to identify feedback loops, which can help explain how systems behave, and in turn how these behaviours lead to particular outcomes associated with problems of interest. These maps comprise a series of theorised causal relations between elements in the system’. |
| CECAN Participatory Systems Mapping (CECAN PSM) | ‘CECAN PSM is similar to other causal systems mapping methods (e.g. CLDs or fuzzy cognitive maps). Its primary purpose is to emphasise and facilitate actionable outcomes from the mapping process by identifying important and controllable components of a system, including those particularly susceptible to change. Of particular importance is that participant-driven applications of network analysis principles are used to identify flows and chains of causal relations, and to develop detailed and meaningful submaps. This helps make sense of typically large and complex maps’. |
| Fuzzy cognitive maps (FCM) | ‘Fuzzy cognitive maps (FCM) illustrate semi-quantitative ‘mental models’ of a system. These maps are made up of elements and connections, which depending on the type of FCM, are assigned different numerical values by those taking part in the mapping process. The factors themselves can be either abstract or measurable. In brief, FCM acknowledges uncertainty in the knowledge of causal processes in complex systems. Their primary purpose is to identify the most influential causal factors, explore what may happen to other factors of a system if something is changed, and examine and compare stakeholders’ ‘mental models’.’ |
| Systems Dynamics | ‘Systems dynamics modelling (SDM) aims to model part of a system that corresponds to a problem, rather than the system as a whole. SDM typically comprises three stages:  1. A CLD (or similar map that includes feedback loops) is used to develop understanding of the structure of the problem system  2. Stock and flow diagrams (S&F) are then used to extend the initial system map and put it into a quantitatively modellable structure. These diagrams conceptualise the behaviour of systems as being the product of the accumulation of stocks (quantity of a variable at a given time) and flows (change in stocks over time), making this form of map distinct from a CLD  3. The S&F diagram is then specified (i.e. quantified) as a set of mathematical formulae known as differential equations, and the simulation (model) is then ‘run’ using computer software’. |
| Bayesian belief networks (BBN) | ‘Bayesian belief networks are graphs informed by the mathematics of Bayesian probability. Their purpose is to capture conditional probabilities of the causal connections between factors in the system, meaning: what are the chances that X happens if Y and Z are true or not? In other words, the ‘belief’ in an outcome is the probability that two or more events may occur simultaneously. A picture of the system is built by connecting various sets of these relations, which can be analysed quantitatively. This approach helps address some of the uncertainty around how different parts of a system interact and influence one another.’ |

**Source of definitions:* Blake, C & Rigby, B et al. (2024) Participatory systems mapping for population health research, policy and practice: guidance on method choice and design. Glasgow, UK: University of Glasgow. Doi.org/10.36399/gla.pubs.316563

**Definition of systems mapping concepts**

| Connections or edges* | ‘The connections between factors in a system map. They are usually drawn as arrows, and typically depict causal relations’. |
| --- | --- |
| Polarity** | ‘A positive (+) or negative (−) sign that indicates the direction of impact of the driving variable on the driven variable. Positive polarity indicates that the impacted variable moves in the same direction (increase or decrease) as the driving variable. Negative polarity indicates that the impacted variable moves in the opposite direction (increase or decrease) to the driving variable. Alternatively, positive link polarity is sometimes indicated by the letter “S” (causing to move in the same direction) and negative link polarity by the letter “O” (causing to move in the opposite direction)’. |
| Delays* | ‘The effect of one factor on another does not necessarily occur immediately, therefore the resultant output lags behind the input’. |
| Strength of influence | The degree to which a map factor can influence another map factor. The strength of influence can be represented visually, for example by varying line thickness or the size of map factors. |
| Conditional probabilities* | ‘The likelihood of an event or outcome occurring, based on the occurrence of a previous event or outcome’. |
| Feedback loops* | ‘When two or more factors interact with each other in the system, such that the effect of the causal impact returns to influence the original cause of said effect, with a reinforcing or dampening impact’. |

Source of definitions:

* Blake, C & Rigby B et al. (2024) Participatory systems mapping for population health research, policy and practice: guidance on method choice and design. Appendices. University of Glasgow. DOI: doi.org/10.36399/gla.pubs.316563

** Ford, D.N. A system dynamics glossary. Syst. Dyn. Rev. 2019; 35: 369-379.
